# Annotated Draft Genome Sequence of the Apple Scab Pathogen Venturia inaequalis

**DOI:** 10.1128/MRA.01062-18

**Published:** 2018-09-27

**Authors:** Thomas A. J. Passey, Andrew D. Armitage, Xiangming Xu

**Affiliations:** a NIAB EMR, East Malling, Kent, United Kingdom; b School of Agriculture, Policy and Development, University of Reading, Reading, United Kingdom; Vanderbilt University

## Abstract

Apple scab is one of the most economically important diseases of apples worldwide. The disease is caused by the haploid ascomycete Venturia inaequalis. We present here an annotated V. inaequalis whole-genome sequence of 72 Mb, assembled into 238 contigs, with 13,761 predicted genes.

## Abstract (precis)

Apple scab is one of the most economically important diseases of apples worldwide. The disease is caused by the haploid ascomycete Venturia inaequalis.

## ANNOUNCEMENT

Venturia inaequalis (phylum Ascomycota, class Dothideomycetes) is the causal agent of apple scab, one of the most important diseases of apples worldwide, and, as a result, has been extensively researched for well over a century (1). If not managed, annual epidemics can result in large numbers of unmarketable fruit. Previously published annotated genome sequences for V. inaequalis have between 1,012 and 1,680 scaffolds (2, 3).

A single-spore isolate of V. inaequalis (05/172) was obtained in 2005 from a lesion on a leaf of Malus x domestica cv. Worcester Pearmain from Ash Farm in Worcestershire, United Kingdom (4). DNA was extracted and sequenced by two methods: (i) DNA was extracted from mycelium using a Qiagen Genomic-tip 100/G kit; the tissue method of sample preparation was used according to the manufacturer’s protocol with options 3B and 4B (adapted to 200 µl proteinase K). Isolation of DNA followed the manufacturer’s protocol with options 5B and 6B. DNA was sent to the Earlham Institute (Norwich, UK), for sequencing using the Pacific Biosciences (PacBio) platform. (ii) DNA of the isolate was extracted for Passey et al. (5). Paired-end genomic libraries were prepared using a NEXTflex Rapid DNA-Seq version 14.02 library prep kit (Bioo Scientific) following the manufacturer’s protocol but modified by using Illumina adapters rather than NEXTflex barcodes. Libraries were validated using a fragment analyzer (Advanced Analytical Technologies), which confirmed a high proportion of library DNA fragments between 600 and 900 bp long. Libraries were sequenced using 2 × 300-bp reads on an Illumina MiSeq platform. Illumina adapters and low-quality base pairs were trimmed from 1,281,750 MiSeq reads with fastq-mcf version 1.04.636 (6).

PacBio sequencing reads (944,907 reads) were corrected, trimmed, and assembled with Canu version 1.2 (7), and the assembly was corrected with MiSeq reads using Pilon version 1.17 (8). Hybrid assembly with both PacBio and MiSeq reads was performed with SPAdes version 3.9.0 (9) and then merged with the Canu assembly using quickmerge version 0.2 (10); the merged assembly was corrected with the MiSeq reads using Pilon. The genome was assembled into 72.3 Mb in 238 contigs (Table 1). Repetitive and low-complexity regions of the merged assembly were identified by repeat masking with RepeatMasker version 4.0.6 (http://www.repeatmasker.org) and TransposonPSI (release 08222010; http://transposonpsi.sourceforge.net), masking 34.2 Mb (47.3%) of the genome, of which 98.7% was due to transposable elements. Quality of the genome assembly was assessed by looking for benchmarking universal single-copy orthologs (BUSCO) with BUSCO version 3 (11) against the Ascomycota odb9 data set, identifying 1,286 (out of 1,315) as present in the assembly. Gene prediction was performed with the use of RNA sequencing (RNA-seq) data from Thakur et al. (12); RNA-seq data were aligned to the genome by STAR version 2.6 (13). A predicted 13,761 genes are present in the assembled genome; 11,597 genes were predicted by Braker1 (14), supplemented by 2,164 genes predicted by CodingQuarry (15) (in pathogen mode) in the intergenic regions of Braker1 gene models. Functional annotation of the genome was performed using Interproscan version 5.18-57.0 (16) and the July 2016 release of the Swiss-Prot database (17).

**TABLE 1:** V. inaequalis isolate 05/172 genome assembly statistics^a^

Statistic	Value for isolate 05/172
No. of contigs	238
Total length (bp)	72,310,420
Largest contig (bp)	3,847,617
GC content (%)	42.75
*N*_50_ (bp)	953,805
*N*_75_ (bp)	531,805
*L*_50_	23
*L*_75_	49

a Assembly produced by merged Canu and SPAdes assemblies using PacBio- and MiSeq-generated sequencing reads.

### Data availability.

The Sequence Read Archive accession numbers are SRR5183052 for the Illumina MiSeq reads and SRR5183051 for the PacBio reads. This whole-genome shotgun project has been deposited at DDBJ/ENA/GenBank under the accession number QFBF00000000 (BioProject number PRJNA354841). The version described in this paper is the first version, QFBF01000000.
